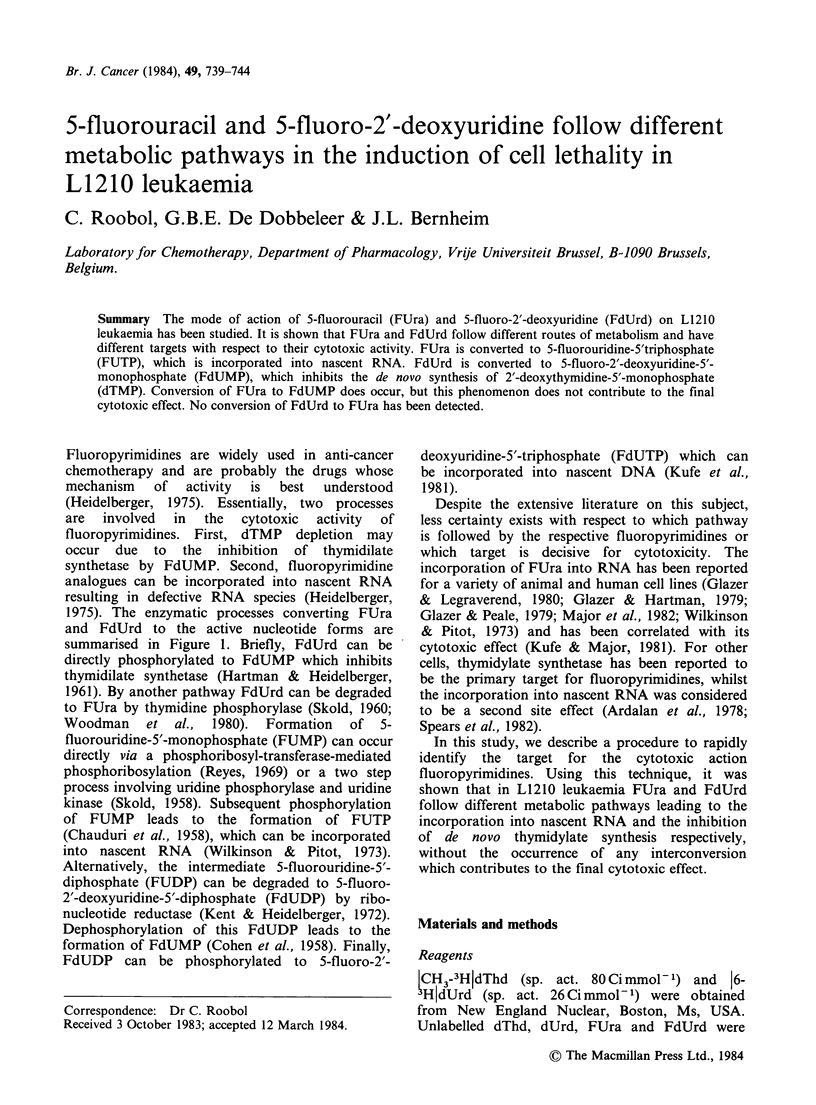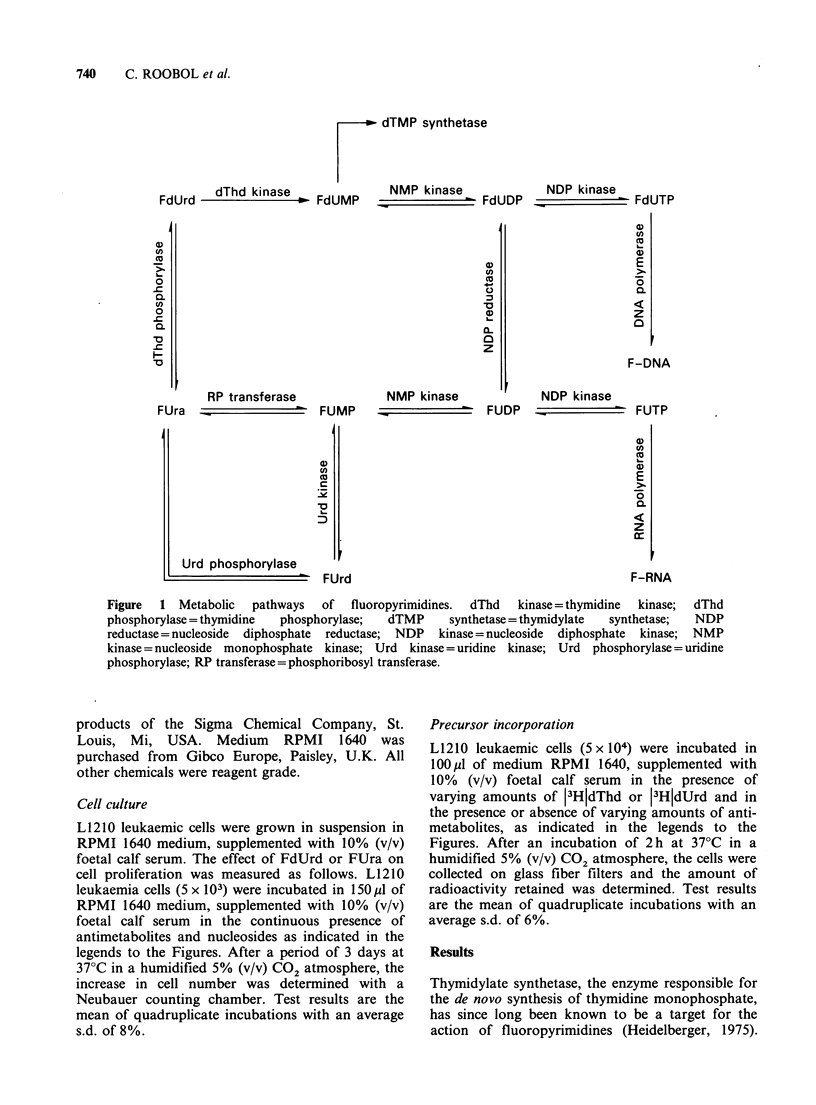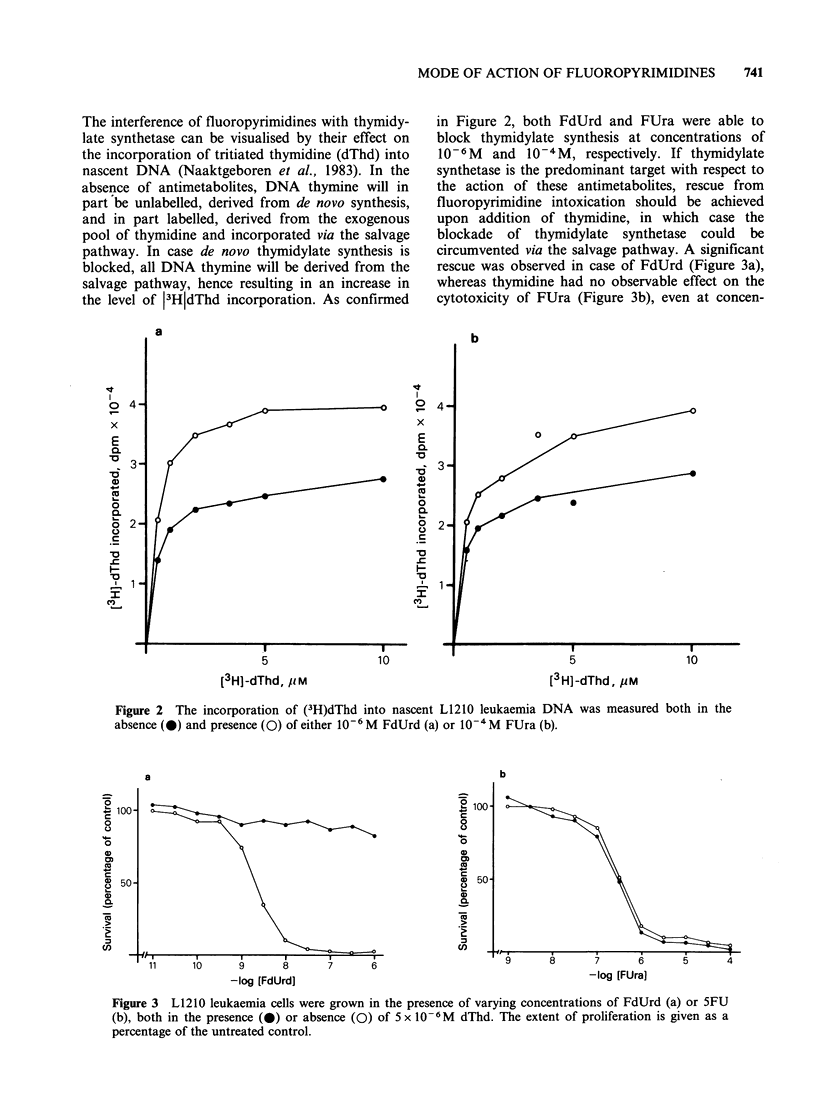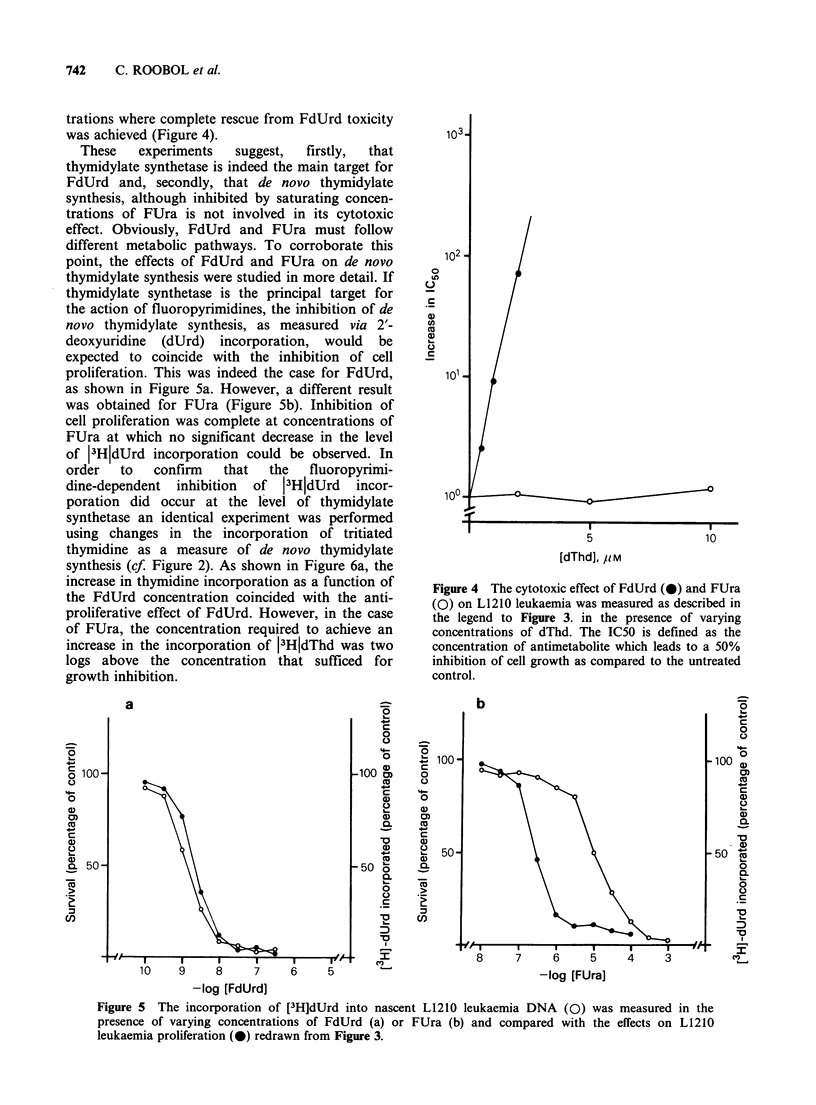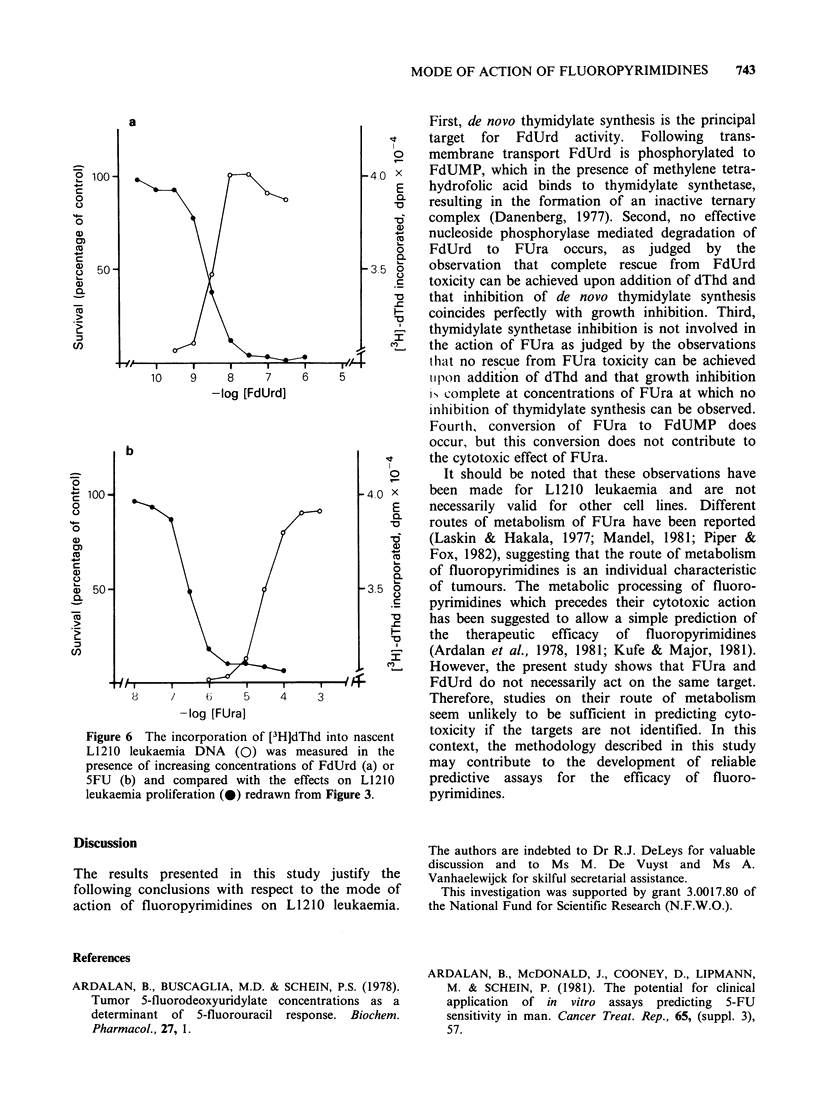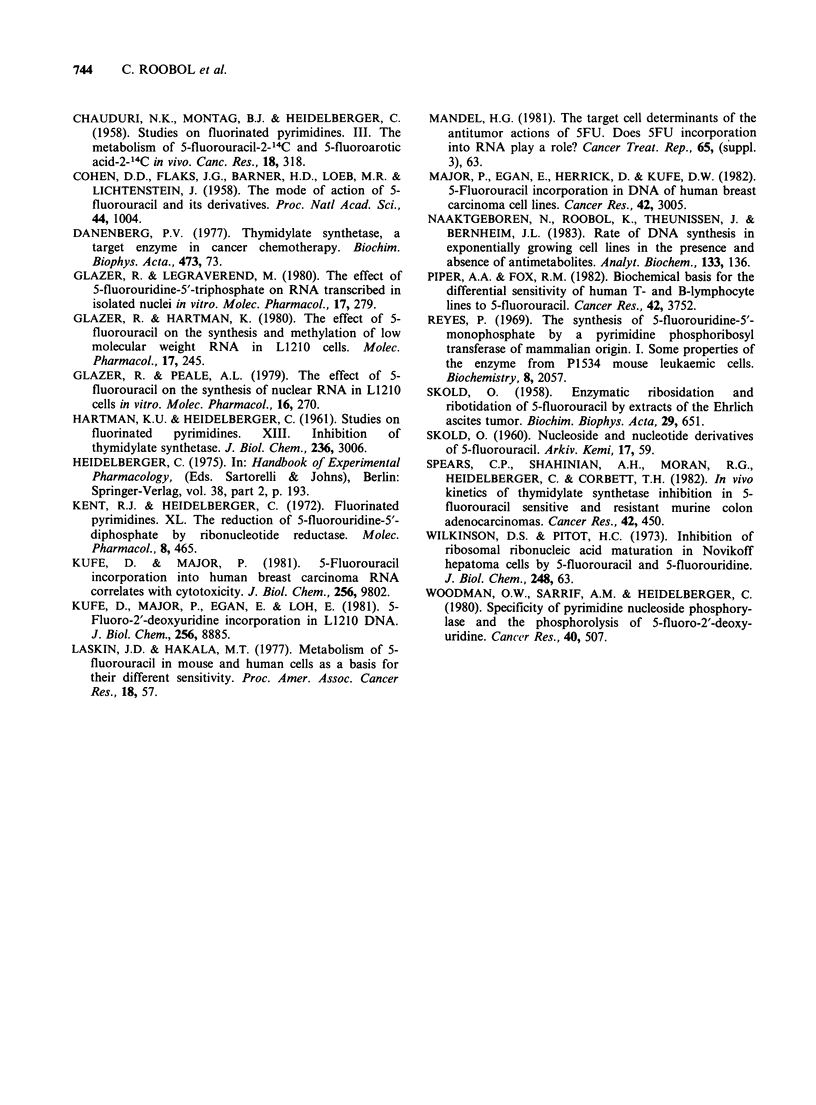# 5-fluorouracil and 5-fluoro-2'-deoxyuridine follow different metabolic pathways in the induction of cell lethality in L1210 leukaemia.

**DOI:** 10.1038/bjc.1984.116

**Published:** 1984-06

**Authors:** C. Roobol, G. B. De Dobbeleer, J. L. Bernheim

## Abstract

The mode of action of 5-fluorouracil (FUra) and 5-fluoro-2'-deoxyuridine (FdUrd) on L1210 leukaemia has been studied. It is shown that FUra and FdUrd follow different routes of metabolism and have different targets with respect to their cytotoxic activity. FUra is converted to 5-fluorouridine-5'triphosphate ( FUTP ), which is incorporated into nascent RNA. FdUrd is converted to 5-fluoro-2'-deoxyuridine-5'-monophosphate (FdUMP), which inhibits the de novo synthesis of 2'-deoxythymidine-5'-monophosphate (dTMP). Conversion of FUra to FdUMP does occur, but this phenomenon does not contribute to the final cytotoxic effect. No conversion of FdUrd to FUra has been detected.


*Br. J. Cancer* (1984), **49**, 739–744

### 5-fluorouracil and 5-fluoro-2′-deoxyuridine follow different metabolic pathways in the induction of cell lethality in L1210 leukaemia

C. Roobol, G.B.E. De Dobbeleer & J.L. Bernheim

*Laboratory for Chemotherapy, Department of Pharmacology, Vrije Universiteit Brussel, B-1090 Brussels, Belgium.*

**Summary** The mode of action of 5-fluorouracil (FUra) and 5-fluoro-2′-deoxyuridine (FdUrd) on L1210 leukaemia has been studied. It is shown that FUra and FdUrd follow different routes of metabolism and have different targets with respect to their cytotoxic activity. FUra is converted to 5-fluorouridine-5′triphosphate (FUTP), which is incorporated into nascent RNA. FdUrd is converted to 5-fluoro-2′-deoxyuridine-5′-monophosphate (FdUMP), which inhibits the *de novo* synthesis of 2′-deoxythymidine-5′-monophosphate (dTMP). Conversion of FUra to FdUMP does occur, but this phenomenon does not contribute to the final cytotoxic effect. No conversion of FdUrd to FUra has been detected.

Fluoropyrimidines are widely used in anti-cancer chemotherapy and are probably the drugs whose mechanism of activity is best understood (Heidelberger, 1975). Essentially, two processes are involved in the cytotoxic activity of fluoropyrimidines. First, dTMP depletion may occur due to the inhibition of thymidilate synthetase by FdUMP. Second, fluoropyrimidine analogues can be incorporated into nascent RNA resulting in defective RNA species (Heidelberger, 1975). The enzymatic processes converting FUra and FdUrd to the active nucleotide forms are summarised in Figure 1. Briefly, FdUrd can be directly phosphorylated to FdUMP which inhibits thymidilate synthetase (Hartman & Heidelberger, 1961). By another pathway FdUrd can be degraded to FUra by thymidine phosphorylase (Skold, 1960; Woodman *et al.*, 1980). Formation of 5-fluorouridine-5′-monophosphate (FUMP) can occur directly *via* a phosphoribosyl-transferase-mediated phosphoribosylation (Reyes, 1969) or a two step process involving uridine phosphorylase and uridine kinase (Skold, 1958). Subsequent phosphorylation of FUMP leads to the formation of FUTP (Chauduri *et al.*, 1958), which can be incorporated into nascent RNA (Wilkinson & Pitot, 1973). Alternatively, the intermediate 5-fluorouridine-5′-diphosphate (FUDP) can be degraded to 5-fluoro-2′-deoxyuridine-5′-diphosphate (FdUDP) by ribonucleotide reductase (Kent & Heidelberger, 1972). Dephosphorylation of this FdUDP leads to the formation of FdUMP (Cohen *et al.*, 1958). Finally, FdUDP can be phosphorylated to 5-fluoro-2′-deoxyuridine-5′-triphosphate (FdUTP) which can be incorporated into nascent DNA (Kufe *et al.*, 1981).

Despite the extensive literature on this subject, less certainty exists with respect to which pathway is followed by the respective fluoropyrimidines or which target is decisive for cytotoxicity. The incorporation of FUra into RNA has been reported for a variety of animal and human cell lines (Glazer & Legraverend, 1980; Glazer & Hartman, 1979; Glazer & Peale, 1979; Major *et al.*, 1982; Wilkinson & Pitot, 1973) and has been correlated with its cytotoxic effect (Kufe & Major, 1981). For other cells, thymidylate synthetase has been reported to be the primary target for fluoropyrimidines, whilst the incorporation into nascent RNA was considered to be a second site effect (Ardalan *et al.*, 1978; Spears *et al.*, 1982).

In this study, we describe a procedure to rapidly identify the target for the cytotoxic action fluoropyrimidines. Using this technique, it was shown that in L1210 leukaemia FUra and FdUrd follow different metabolic pathways leading to the incorporation into nascent RNA and the inhibition of *de novo* thymidylate synthesis respectively, without the occurrence of any interconversion which contributes to the final cytotoxic effect.

### Materials and methods

### *Reagents*

|$CH_3$-$^3H$|dThd (sp. act. 80 Ci mmol$^{-1}$) and |6-$^3H$|dUrd (sp. act. 26 Ci mmol$^{-1}$) were obtained from New England Nuclear, Boston, Ms, USA. Unlabelled dThd, dUrd, FUra and FdUrd were

Correspondence: Dr C. Roobol
Received 3 October 1983; accepted 12 March 1984.

© The Macmillan Press Ltd., 1984

**Figure 1** Metabolic pathways of fluoropyrimidines. dThd kinase = thymidine kinase; dThd phosphorylase = thymidine phosphorylase; dTMP synthetase = thymidylate synthetase; NDP reductase = nucleoside diphosphate reductase; NDP kinase = nucleoside diphosphate kinase; NMP kinase = nucleoside monophosphate kinase; Urd kinase = uridine kinase; Urd phosphorylase = uridine phosphorylase; RP transferase = phosphoribosyl transferase.

products of the Sigma Chemical Company, St. Louis, Mi, USA. Medium RPMI 1640 was purchased from Gibco Europe, Paisley, U.K. All other chemicals were reagent grade.

*Cell culture*

L1210 leukaemic cells were grown in suspension in RPMI 1640 medium, supplemented with 10% (v/v) foetal calf serum. The effect of FdUrd or FUra on cell proliferation was measured as follows. L1210 leukaemia cells ($5 \times 10^3$) were incubated in 150 $\mu$l of RPMI 1640 medium, supplemented with 10% (v/v) foetal calf serum in the continuous presence of antimetabolites and nucleosides as indicated in the legends to the Figures. After a period of 3 days at 37°C in a humidified 5% (v/v) $CO_2$ atmosphere, the increase in cell number was determined with a Neubauer counting chamber. Test results are the mean of quadruplicate incubations with an average s.d. of 8%.

*Precursor incorporation*

L1210 leukaemic cells ($5 \times 10^4$) were incubated in 100 $\mu$l of medium RPMI 1640, supplemented with 10% (v/v) foetal calf serum in the presence of varying amounts of $|^3H|$dThd or $|^3H|$dUrd and in the presence or absence of varying amounts of antimetabolites, as indicated in the legends to the Figures. After an incubation of 2 h at 37°C in a humidified 5% (v/v) $CO_2$ atmosphere, the cells were collected on glass fiber filters and the amount of radioactivity retained was determined. Test results are the mean of quadruplicate incubations with an average s.d. of 6%.

### Results

Thymidylate synthetase, the enzyme responsible for the *de novo* synthesis of thymidine monophosphate, has since long been known to be a target for the action of fluoropyrimidines (Heidelberger, 1975).

The interference of fluoropyrimidines with thymidylate synthetase can be visualised by their effect on the incorporation of tritiated thymidine (dThd) into nascent DNA (Naaktgeboren *et al.*, 1983). In the absence of antimetabolites, DNA thymine will in part be unlabelled, derived from *de novo* synthesis, and in part labelled, derived from the exogenous pool of thymidine and incorporated *via* the salvage pathway. In case *de novo* thymidylate synthesis is blocked, all DNA thymine will be derived from the salvage pathway, hence resulting in an increase in the level of $|^3H|$dThd incorporation. As confirmed in Figure 2, both FdUrd and FUra were able to block thymidylate synthesis at concentrations of $10^{-6}$M and $10^{-4}$M, respectively. If thymidylate synthetase is the predominant target with respect to the action of these antimetabolites, rescue from fluoropyrimidine intoxication should be achieved upon addition of thymidine, in which case the blockade of thymidylate synthetase could be circumvented *via* the salvage pathway. A significant rescue was observed in case of FdUrd (Figure 3a), whereas thymidine had no observable effect on the cytotoxicity of FUra (Figure 3b), even at concen-

**Figure 2** The incorporation of ($^3$H)dThd into nascent L1210 leukaemia DNA was measured both in the absence (●) and presence (○) of either $10^{-6}$ M FdUrd (a) or $10^{-4}$ M FUra (b).

**Figure 3** L1210 leukaemia cells were grown in the presence of varying concentrations of FdUrd (a) or 5FU (b), both in the presence (●) or absence (○) of $5\times10^{-6}$M dThd. The extent of proliferation is given as a percentage of the untreated control.

trations where complete rescue from FdUrd toxicity was achieved (Figure 4).

These experiments suggest, firstly, that thymidylate synthetase is indeed the main target for FdUrd and, secondly, that *de novo* thymidylate synthesis, although inhibited by saturating concentrations of FUra is not involved in its cytotoxic effect. Obviously, FdUrd and FUra must follow different metabolic pathways. To corroborate this point, the effects of FdUrd and FUra on *de novo* thymidylate synthesis were studied in more detail. If thymidylate synthetase is the principal target for the action of fluoropyrimidines, the inhibition of *de novo* thymidylate synthesis, as measured *via* 2′-deoxyuridine (dUrd) incorporation, would be expected to coincide with the inhibition of cell proliferation. This was indeed the case for FdUrd, as shown in Figure 5a. However, a different result was obtained for FUra (Figure 5b). Inhibition of cell proliferation was complete at concentrations of FUra at which no significant decrease in the level of [$^{3}$H]dUrd incorporation could be observed. In order to confirm that the fluoropyrimidine-dependent inhibition of [$^{3}$H]dUrd incorporation did occur at the level of thymidylate synthetase an identical experiment was performed using changes in the incorporation of tritiated thymidine as a measure of *de novo* thymidylate synthesis (*cf.* Figure 2). As shown in Figure 6a, the increase in thymidine incorporation as a function of the FdUrd concentration coincided with the anti-proliferative effect of FdUrd. However, in the case of FUra, the concentration required to achieve an increase in the incorporation of [$^{3}$H]dThd was two logs above the concentration that sufficed for growth inhibition.

**Figure 4** The cytotoxic effect of FdUrd (●) and FUra (○) on L1210 leukaemia was measured as described in the legend to **Figure** 3. in the presence of varying concentrations of dThd. The IC50 is defined as the concentration of antimetabolite which leads to a 50% inhibition of cell growth as compared to the untreated control.

**Figure 5** The incorporation of [$^{3}$H]dUrd into nascent L1210 leukaemia DNA (○) was measured in the presence of varying concentrations of FdUrd (a) or FUra (b) and compared with the effects on L1210 leukaemia proliferation (●) redrawn from **Figure** 3.

**Figure 6** The incorporation of [$^3$H]dThd into nascent L1210 leukaemia DNA (○) was measured in the presence of increasing concentrations of FdUrd (a) or 5FU (b) and compared with the effects on L1210 leukaemia proliferation (●) redrawn from **Figure 3**.

### Discussion

The results presented in this study justify the following conclusions with respect to the mode of action of fluoropyrimidines on L1210 leukaemia. First, *de novo* thymidylate synthesis is the principal target for FdUrd activity. Following trans-membrane transport FdUrd is phosphorylated to FdUMP, which in the presence of methylene tetra-hydrofolic acid binds to thymidylate synthetase, resulting in the formation of an inactive ternary complex (Danenberg, 1977). Second, no effective nucleoside phosphorylase mediated degradation of FdUrd to FUra occurs, as judged by the observation that complete rescue from FdUrd toxicity can be achieved upon addition of dThd and that inhibition of *de novo* thymidylate synthesis coincides perfectly with growth inhibition. Third, thymidylate synthetase inhibition is not involved in the action of FUra as judged by the observations that no rescue from FUra toxicity can be achieved upon addition of dThd and that growth inhibition is complete at concentrations of FUra at which no inhibition of thymidylate synthesis can be observed. Fourth, conversion of FUra to FdUMP does occur, but this conversion does not contribute to the cytotoxic effect of FUra.

It should be noted that these observations have been made for L1210 leukaemia and are not necessarily valid for other cell lines. Different routes of metabolism of FUra have been reported (Laskin & Hakala, 1977; Mandel, 1981; Piper & Fox, 1982), suggesting that the route of metabolism of fluoropyrimidines is an individual characteristic of tumours. The metabolic processing of fluoro-pyrimidines which precedes their cytotoxic action has been suggested to allow a simple prediction of the therapeutic efficacy of fluoropyrimidines (Ardalan *et al.*, 1978, 1981; Kufe & Major, 1981). However, the present study shows that FUra and FdUrd do not necessarily act on the same target. Therefore, studies on their route of metabolism seem unlikely to be sufficient in predicting cyto-toxicity if the targets are not identified. In this context, the methodology described in this study may contribute to the development of reliable predictive assays for the efficacy of fluoro-pyrimidines.

The authors are indebted to Dr R.J. DeLeys for valuable discussion and to Ms M. De Vuyst and Ms A. Vanhaelewijck for skilful secretarial assistance.

This investigation was supported by grant 3.0017.80 of the National Fund for Scientific Research (N.F.W.O.).

### References

ARDALAN, B., BUSCAGLIA, M.D. & SCHEIN, P.S. (1978). Tumor 5-fluorodeoxyuridylate concentrations as a determinant of 5-fluorouracil response. *Biochem. Pharmacol.*, **27**, 1.

ARDALAN, B., McDONALD, J., COONEY, D., LIPMANN, M. & SCHEIN, P. (1981). The potential for clinical application of *in vitro* assays predicting 5-FU sensitivity in man. *Cancer Treat. Rep.*, **65**, (suppl. 3), 57.

CHAUDURI, N.K., MONTAG, B.J. & HEIDELBERGER, C. (1958). Studies on fluorinated pyrimidines. III. The metabolism of 5-fluorouracil-2-$^{14}$C and 5-fluoroarotic acid-2-$^{14}$C *in vivo*. *Canc. Res.*, **18**, 318.

COHEN, D.D., FLAKS, J.G., BARNER, H.D., LOEB, M.R. & LICHTENSTEIN, J. (1958). The mode of action of 5-fluorouracil and its derivatives. *Proc. Natl Acad. Sci.*, **44**, 1004.

DANENBERG, P.V. (1977). Thymidylate synthetase, a target enzyme in cancer chemotherapy. *Biochim. Biophys. Acta.*, **473**, 73.

GLAZER, R. & LEGRAVEREND, M. (1980). The effect of 5-fluorouridine-5′-triphosphate on RNA transcribed in isolated nuclei *in vitro*. *Molec. Pharmacol.*, **17**, 279.

GLAZER, R. & HARTMAN, K. (1980). The effect of 5-fluorouracil on the synthesis and methylation of low molecular weight RNA in L1210 cells. *Molec. Pharmacol.*, **17**, 245.

GLAZER, R. & PEALE, A.L. (1979). The effect of 5-fluorouracil on the synthesis of nuclear RNA in L1210 cells *in vitro*. *Molec. Pharmacol.*, **16**, 270.

HARTMAN, K.U. & HEIDELBERGER, C. (1961). Studies on fluorinated pyrimidines. XIII. Inhibition of thymidylate synthetase. *J. Biol. Chem.*, **236**, 3006.

HEIDELBERGER, C. (1975). In: *Handbook of Experimental Pharmacology*, (Eds. Sartorelli & Johns), Berlin: Springer-Verlag, vol. 38, part 2, p. 193.

KENT, R.J. & HEIDELBERGER, C. (1972). Fluorinated pyrimidines. XL. The reduction of 5-fluorouridine-5′-diphosphate by ribonucleotide reductase. *Molec. Pharmacol.*, **8**, 465.

KUFE, D. & MAJOR, P. (1981). 5-Fluorouracil incorporation into human breast carcinoma RNA correlates with cytotoxicity. *J. Biol. Chem.*, **256**, 9802.

KUFE, D., MAJOR, P., EGAN, E. & LOH, E. (1981). 5-Fluoro-2′-deoxyuridine incorporation in L1210 DNA. *J. Biol. Chem.*, **256**, 8885.

LASKIN, J.D. & HAKALA, M.T. (1977). Metabolism of 5-fluorouracil in mouse and human cells as a basis for their different sensitivity. *Proc. Amer. Assoc. Cancer Res.*, **18**, 57.

MANDEL, H.G. (1981). The target cell determinants of the antitumor actions of 5FU. Does 5FU incorporation into RNA play a role? *Cancer Treat. Rep.*, **65**, (suppl. 3), 63.

MAJOR, P., EGAN, E., HERRICK, D. & KUFE, D.W. (1982). 5-Fluorouracil incorporation in DNA of human breast carcinoma cell lines. *Cancer Res.*, **42**, 3005.

NAAKTGEBOREN, N., ROOBOL, K., THEUNISSEN, J. & BERNHEIM, J.L. (1983). Rate of DNA synthesis in exponentially growing cell lines in the presence and absence of antimetabolites. *Analyt. Biochem.*, **133**, 136.

PIPER, A.A. & FOX, R.M. (1982). Biochemical basis for the differential sensitivity of human T- and B-lymphocyte lines to 5-fluorouracil. *Cancer Res.*, **42**, 3752.

REYES, P. (1969). The synthesis of 5-fluorouridine-5′-monophosphate by a pyrimidine phosphoribosyl transferase of mammalian origin. I. Some properties of the enzyme from P1534 mouse leukaemic cells. *Biochemistry*, **8**, 2057.

SKOLD, O. (1958). Enzymatic ribosidation and ribotidation of 5-fluorouracil by extracts of the Ehrlich ascites tumor. *Biochim. Biophys. Acta*, **29**, 651.

SKOLD, O. (1960). Nucleoside and nucleotide derivatives of 5-fluorouracil. *Arkiv. Kemi*, **17**, 59.

SPEARS, C.P., SHAHINIAN, A.H., MORAN, R.G., HEIDELBERGER, C. & CORBETT, T.H. (1982). *In vivo* kinetics of thymidylate synthetase inhibition in 5-fluorouracil sensitive and resistant murine colon adenocarcinomas. *Cancer Res.*, **42**, 450.

WILKINSON, D.S. & PITOT, H.C. (1973). Inhibition of ribosomal ribonucleic acid maturation in Novikoff hepatoma cells by 5-fluorouracil and 5-fluorouridine. *J. Biol. Chem.*, **248**, 63.

WOODMAN, O.W., SARRIF, A.M. & HEIDELBERGER, C. (1980). Specificity of pyrimidine nucleoside phosphorylase and the phosphorolysis of 5-fluoro-2′-deoxyuridine. *Cancer Res.*, **40**, 507.